# Accelerated Telomere Shortening in Acromegaly; IGF-I Induces Telomere Shortening and Cellular Senescence

**DOI:** 10.1371/journal.pone.0140189

**Published:** 2015-10-08

**Authors:** Ryusaku Matsumoto, Hidenori Fukuoka, Genzo Iguchi, Yukiko Odake, Kenichi Yoshida, Hironori Bando, Kentaro Suda, Hitoshi Nishizawa, Michiko Takahashi, Shozo Yamada, Wataru Ogawa, Yutaka Takahashi

**Affiliations:** 1 Division of Diabetes and Endocrinology, Department of Internal Medicine, Kobe University Graduate School of Medicine, Kobe, Hyogo, Japan; 2 Division of Diabetes and Endocrinology, Kobe University Hospital, Kobe, Hyogo, Japan; 3 Department of Hypothalamic and Pituitary Surgery, Toranomon Hospital, Minato-ku, Tokyo, Japan; University of Newcastle, UNITED KINGDOM

## Abstract

**Objective:**

Patients with acromegaly exhibit reduced life expectancy and increased prevalence of age-related diseases, such as diabetes, hypertension, and cardiovascular disease. However, the underlying mechanism has not been fully elucidated. Telomere shortening is reportedly associated with reduced life expectancy and increased prevalence of these age-related diseases.

**Methods:**

We measured telomere length in patients with acromegaly using quantitative PCR method. The effect of GH and IGF-I on telomere length and cellular senescence was examined in human skin fibroblasts.

**Results:**

Patients with acromegaly exhibited shorter telomere length than age-, sex-, smoking-, and diabetes-matched control patients with non-functioning pituitary adenoma (0.62 ± 0.23 vs. 0.75 ± 0.35, respectively, *P* = 0.047). In addition, telomere length in acromegaly was negatively correlated with the disease duration (*R* ^*2*^ = 0.210, *P* = 0.003). *In vitro* analysis revealed that not GH but IGF-I induced telomere shortening in human skin fibroblasts. Furthermore, IGF-I-treated cells showed increased senescence-associated β-galactosidase activity and expression of p53 and p21 protein. IGF-I-treated cells reached the Hayflick limit earlier than GH- or vehicle-treated cells, indicating that IGF-I induces cellular senescence.

**Conclusion:**

Shortened telomeres in acromegaly and cellular senescence induced by IGF-I can explain, in part, the underlying mechanisms by which acromegaly exhibits an increased morbidity and mortality in association with the excess secretion of IGF-I.

## Introduction

Telomeres consist of repetitive DNA sequences, thousands of “TTAGGG” tandem repeats, which are located at the ends of linear chromosomes in most somatic cells [1]. Telomere ends form a cap-like structure to protect the ends of chromosomes from degeneration and fusion [2]. However, telomeres shorten during each cell division and when they reach a critically short length, cell cycle arrest and senescence occur; this is known as the “Hayflick limit” in cultured human cells [3]. Telomere damage activates DNA damage response (DDR), a signaling pathway in which cell cycle progression is blocked via an increased production of p53 and cyclin-dependent kinase (Cdk) inhibitor p21 protein [4]. DDR subsequently induces cellular senescence.

A number of observations suggest a close connection between telomere length and mortality and age-related disease [5]. Telomere length measured in peripheral leukocytes is related to mortality; subjects with shorter telomeres are more likely to succumb to cardiovascular disease and infectious diseases [6]. Furthermore, exposure to various stresses and age-related diseases such as diabetes, cardiovascular disease, and neurodegenerative disease are associated with shortened telomeres [7–9]. Smoking, obesity, hypertension, and atherosclerosis are also associated with shortened telomeres [10–12]. As an underlying mechanism, it has been reported that the increased oxidative stress enhances telomere DNA damage. Telomeres are rich in guanine residues and may be particularly sensitive to reactive oxygen species (ROS) because guanine can be oxidized to 8-hydroxyguanine, which is unstable [5].

Recent studies have focused on the relationship between telomere length and endocrine disorders. Patients with polycystic ovary syndrome reportedly exhibit a shortened telomere length [13]. Aulinas et al. reported that patients with active Cushing’s syndrome showed shortened telomeres [14]. Although the potential relationship between telomere length and the growth hormone (GH) and insulin-like growth factor-I (IGF-I) axis has been discussed [15], to the best of our knowledge, telomere length in acromegalic patients has not been reported. Acromegaly is characterized by the over-secretion of GH, mostly caused by GH-producing pituitary adenomas. It is well known that patients with acromegaly have increased mortality, which is associated with comorbidities of age-related disease such as cardiovascular, cerebrovascular, respiratory, and malignant diseases [16, 17]. Although the increased morbidity and mortality is strongly associated with the degree of GH and IGF-I excess, the precise underlying mechanisms have not been fully elucidated.

Here, we examined the telomere length in peripheral leukocytes in patients with acromegaly and control patients with non-functioning pituitary adenoma (NFPA). In addition, we investigated the effect of GH and IGF-I on telomere length and cellular senescence to clarify the underlying mechanisms.

## Materials and Methods

### Patients

This study was approved by the Kobe University Hospital and Toranomon Hospital Ethics Committee and written informed consent was obtained from all subjects. We recruited 61 consecutive patients with acromegaly and 27 consecutive patients with NFPA who underwent transsphenoidal surgery at Toranomon Hospital between 2005 and 2010. The diagnosis of acromegaly and NFPA was based on their clinical findings, laboratory data, and imaging studies, and confirmed by pathological findings in the surgically removed tumors. The clinical data and blood samples for telomere length analysis were obtained before the surgery, thus all patients with acromegaly had an active disease. Seven patients with acromegaly received pre-operative medical therapies (5 patients received somatostatin analogue, 1 patient received dopamine agonist, and 1 patient received both of them) and no one received radiotherapy. We excluded patients who had undergone previous pituitary surgery, patients with malignancy and other endocrine disorders, and patients in whom hormone replacement therapy (hydrocortisone, thyroxin, GH, gonadotropins, and/or gonadal steroid hormones) was necessary before surgery. We also excluded patients with NFPA whose IGF-I standard deviation score (SDS) was lower than −2.0.

### Endocrinological evaluation

Endocrinological data were obtained before the surgery. The diagnosis of acromegaly was based on clinical signs, lack of serum GH suppression to < 1 ng/mL during a 75 g oral glucose tolerance test (OGTT), elevated serum IGF-I levels corresponding to the normal range for age- and sex-matched individuals, and the presence of pituitary tumors [18]. The duration of the disease was assessed visually by comparison of photographs and by the onset of related symptoms as previously described [18]. All patients underwent transsphenoidal surgery that yielded a histological diagnosis of GH-producing pituitary adenoma or NFPA. Clinical data were retrospectively collected from patients’ medical records. Basal serum levels of GH and IGF-I were measured in the morning after overnight fasting. Serum GH and IGF-I levels were measured by an immunoenzymometric assay using the ST AIA-PACK hGH kit (TOSOH Corporation, Tokyo, Japan) and an immunoradiometric assay using “Daiichi” IGF-I IRMA kit (FUJIFILM RI Pharma Co.,Tokyo, Japan), respectively. Intra- and inter-assay coefficients of variation (C.V.) for the assay of GH and IGF-I were as follows: GH (intra-C.V. 1.3% and inter-C.V. 3.3%) and IGF-I (intra-C.V. 1.1% and inter-C.V. 2.2%), respectively.

### Histological analysis

Surgically removed adenoma tissues were fixed in formaldehyde, embedded in paraffin, and cut into 3 μm thick sections for immunohistological staining. For GH immunostaining, anti-GH polyclonal antibody was used (Dako, Carpinteria, CA, USA; A0570). The diagnosis of acromegaly or NFPA was confirmed by the histological findings. NFPA was defined as an adenoma in which GH, PRL, ACTH, and TSH were negative.

### Telomere length measurement

Leukocyte telomere length was examined in blood samples, which were collected before the surgery. Genomic DNA was extracted from peripheral leukocytes using the Gentra Puregene Blood Kit (QIAGEN, Venlo, Netherlands). The telomere length for each patient was determined using a quantitative PCR assay as previously described [19] with a slight modification. All assays were performed by the Step One Plus^TM^ Real Time PCR system (Applied Biosystems, Tokyo, Japan) in a 96-well plate. Twenty ng of DNA was subjected to the PCR reaction. Each reaction well included 1× SYBR Premix Ex Taq^TM^ II (TaKaRa Bio Inc., Shiga, Japan), 1.5 mM MgCl_2_, 1 mM dithiothreitol, and 1 M betaine. The telomere quantity was normalized to β-globin gene. Each primer sequence and concentration in the reaction are shown in S1 Table. The thermal cycling profile was 15 min at 95°C; two repeats of 15 s at 94°C followed by 15 s at 49°C; 36 repeats of 15 s at 95°C, 120 s at 58°C, and 30 s at 74°C; followed by a melting curve analysis for verification of the PCR product. All samples were assayed in triplicate using a standard curve with 5 concentrations spanning an 81-fold range (100, 33.3, 11.1, 3.7, 1.23 ng) of standard DNA (obtained from a healthy 30-year-old man). The relative telomere length (RTL) was calculated as the ratio of the telomere repeat copy number to the single gene copy number (T/S) according to the standard curve. Two patients with RTLs over 2.0 were excluded from subsequent statistical analysis because they were outliers (a 57-year-old female in the Acro group with an RTL of 2.1, and a 41-year-old female in the NFPA group with an RTL of 2.1).

### Cell culture and GH/IGF-I treatment

Human skin fibroblasts were obtained from a 17-year-old healthy man after obtaining written informed consent. Cells were cultured in Dulbecco's modified Eagle's medium containing 10% fetal bovine serum (FBS) and incubated at 37°C in a humidified atmosphere of 5% CO_2_ and 95% air. Cells were treated with 100 or 500 ng/mL of recombinant human GH (Eli Lilly, Kobe, Japan), recombinant human IGF-I (Astellas Pharma Inc. Tokyo, Japan), or vehicle. The medium with these compounds was changed every other day. Cultured cells were passaged before reaching confluence and seeded at 3.5 × 10^3^ cells/cm^2^. Population doubling levels (PDL) were calculated as PDL = log_2_(*N*_*n*_/*N*_*0*_), where *N*_*n*_ is the cell number at the passage and *N*_*0*_ is the initial number of fibroblasts.

### Quantitative reverse transcription PCR

Human fibroblasts at day 10 and 27 (PDL of 10–11 and 21–22) were used for these experiments (S1A Fig). The mRNA expression levels of p53 and p21 were quantified using qRT-PCR at day 10 and 27. That of Interleukine-6 (IL-6) was quantified at day27. Total RNA was extracted from the cells using TRI reagent (Molecular Research Center, Inc., OH, USA). Five-hundred ng of total RNA was subjected to reverse transcription using the ReverTra Ace qPCR RT Kit (TOYOBO, Osaka, Japan). All quantitative PCR reactions were performed with the Step One Plus^TM^ Real Time PCR system (Applied Biosystems, Tokyo, Japan) using SYBR mix Ex Taq^TM^ II (TaKaRa Bio Inc., Shiga, Japan). The thermal cycling profiles were as follows: initial denaturation at 95°C for 15 min, followed by 30 cycles of denaturation at 94°C for 15 sec, annealing at 55°C for 15 sec, and extension at 72°C for 15 sec. β-actin was used as an internal control. Each primer sequence used in the experiments is shown in S2 Table. All samples were assayed in duplicate. The representative results from four independent experiments are shown.

### Immunoblotting

Human fibroblasts at day 10 and 27 (PDL of 10–11 and 21–22) were used for these experiments (S1A Fig). Cells were washed twice with PBS and lysed in lysis buffer containing 50 mM Tris HCl pH 7.5, 30 mM KCl, 5 mM EDTA, 1% NP-40, 1 mM dithiothreitol, and 0.1% sodium dodecyl sulfate (SDS), protease inhibitor (Nacalai Tesque, Kyoto, Japan), and phosphatase inhibitor (Pierce Thermo Fisher Scientific, IL, USA). Twenty μg of protein per lane was subjected to SDS-polyacrylamide gel electrophoresis (PAGE), and then transferred to a polyvinylidene fluoride membrane. The membrane was incubated with primary antibody overnight at 4°C. Antibodies for total p53 and phosphorylated p53 (Ser 15) were obtained from Cell Signaling Technology (Danvers, MA, USA). Anti-p21 antibody and anti-β-actin antibody were obtained from Santa Cruz Biotechnology (Dallas, TX, USA) and Sigma-Aldrich (St Louis, MO, USA), respectively. After incubation with horseradish peroxidase-conjugated secondary antibody for 1 hr at room temperature, signals were visualized with ImmunoStar LD (WAKO, Tokyo, Japan) or Chemi-Lumi One L solution (Nacalai Tesque, Kyoto, Japan).

### Senescence-associated β-galactosidase staining

Senescence-associated β-galactosidase staining (SA β-Gal) was performed as previously described with a slight modification [20]. Human fibroblasts at day 38 (PDL of 28–29) were used for these experiments (S1A Fig). Briefly, cells were washed with PBS and fixed with 0.5% glutaraldehyde solution for 15 min. The cells were washed again two times with pH 5.5 PBS with 40 mM MgCl_2_. Subsequently, staining solution (1 mg/mL 5-bromo-4-chloro-3-indolyl β-d-galactosidase (X-gal) in dimethylformamide, 5 mM potassium ferrocyanide, and 5 mM potassium ferricyanide dissolved in pH 5.5 PBS with 40 mM MgCl_2_) was added and cells were incubated at 37°C for 8 hrs. After incubation, the cells were washed with PBS and photographed. The proportion of senescent cells stained blue was determined in 6 fields at 200× magnification.

### Statistical analysis

Data are appropriately expressed as means ± standard deviations or medians [interquartile range]. Continuous data were compared by a Student’s *t*-test or a Mann—Whitney test and categorical data were compared by a χ^2^ test or Fischer’s exact test, as appropriate. Multiple groups were compared by a one-way analysis of variance with a post-hoc Fischer’s least significant difference test, a Kruskal—Wallis test with a post-hoc Scheffe test, or a χ^2^ test followed by Tukey’s honestly significant difference test, as appropriate. Homogeneity of variance was tested by Levene’s test. Pearson’s test was used to evaluate the correlation between two variables. *P*-values of 0.05 or less were considered significant. Statistical analyses were performed using JMP Statistical Database Software version 8. 0. 1 (SAS Institute, Inc. Cary, NC, USA).

## Results

### Telomere length in acromegaly

**Fig 1:**
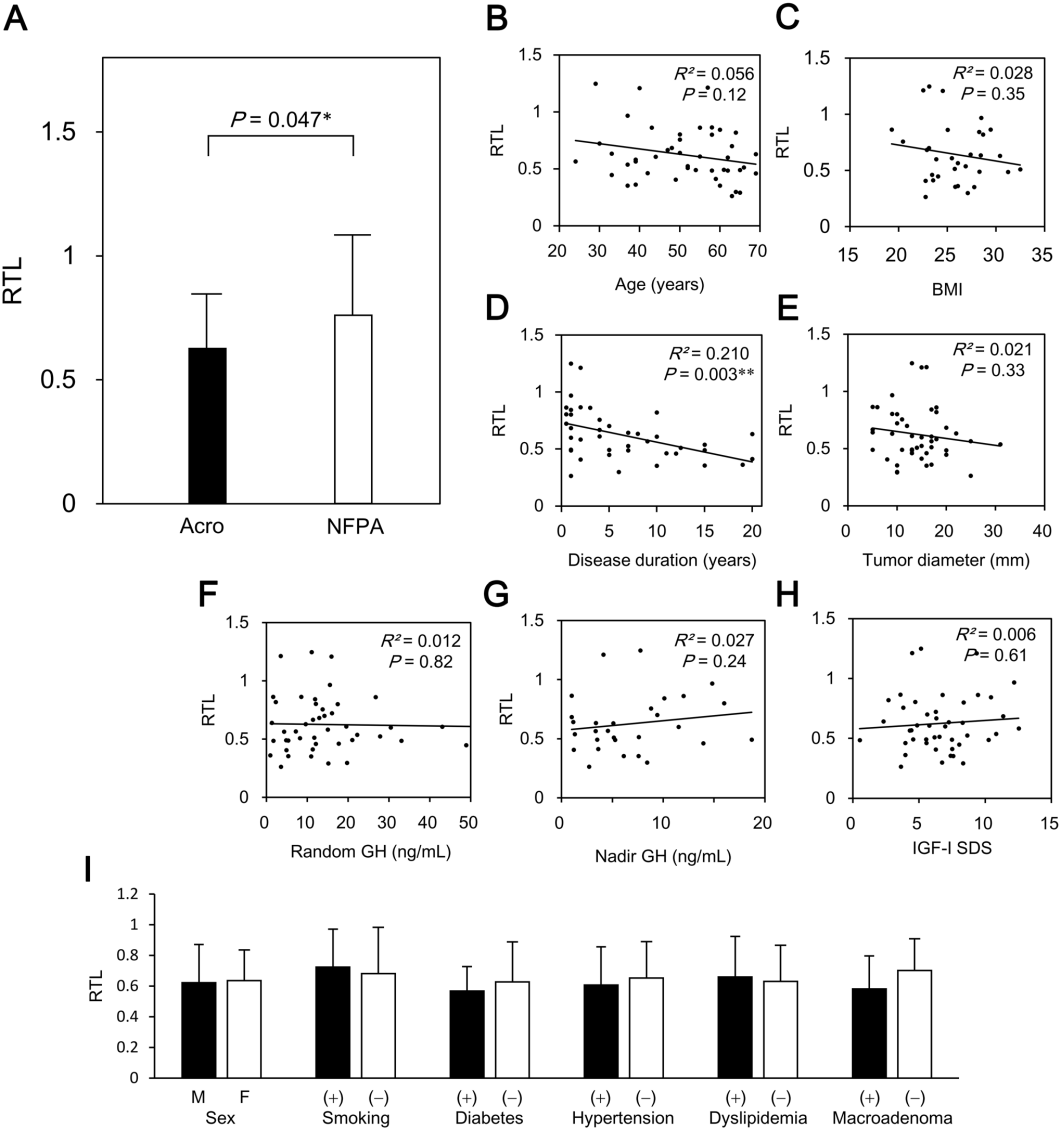
Telomere length in patients with acromegaly. **A,** Comparison of relative telomere length between patients with acromegaly and non-functioning pituitary adenoma. **B–H**, Correlations between relative telomere length and clinical indices (age, BMI, disease duration, tumor diameter, tandom GH, nadir GH during OGTT, and IGF-I SDS). **I**, Comparison of relative telomere length for the clinical indices (sex, hypertension, diabetes, dyslipidemia, macroadenoma) in patients with acromegaly. Relative telomere length was compared using Student’s *t*-tests. Data are expressed as median with interquartile range. *P < 0.05, **P < 0.01. RTL, relative telomere length; Acro, acromegaly; NFPA, non-functioning pituitary adenoma.

To investigate whether patients with acromegaly exhibit shortened telomeres, we compared them with age-, sex-, smoking-, and diabetes-matched patients with NFPA. Forty-six patients with acromegaly after the exclusion of the patients who met the exclusion criteria (the Acro group) and 20 out of 27 patients with NFPA (the NFPA group) were enrolled in this study. The clinical characteristics of these groups are shown in Table 1. As expected for the initial matching, there were no significant differences in age, sex ratio, and the ratio of smoking and diabetes between these two groups. Also, there were no statistical differences in BMI, HbA1c levels and the prevalence of hypertension and dyslipidemia. All patients in the NFPA group had a macroadenoma and the tumor diameter was significantly greater in the NFPA group than in the Acro group. As predicted, the preoperative serum random GH levels and IGF-I SDS were significantly higher in the Acro group than in the NFPA group. With respect to other pituitary hormones, although no significant differences were observed, TSH levels tended to be lower in the Acro group. RTL was evaluated in these groups. As shown in Fig 1A, RTL in the Acro group was significantly shorter than that in the NFPA group (0.62 ± 0.23 vs. 0.75 ± 0.35, *P* = 0.047).

**Table 1:** Clinical characteristics of the acromegaly (Acro) and the non-functioning pituitary adenoma (NFPA) group.

	Acro (n = 46)	NFPA (n = 20)	*P*
Sex (male/female)	11/35	8/12	0.16
Age (years)	50.9 ± 12.0	56.2 ± 12.2	0.12
BMI	25.7 ± 3.0	25.3 ± 2.1	0.83
Smoking (%)	15.2	25.0	0.41
Diabetes (%)	19.4	7.8	0.33
HbA1c (%)	5.9 ± 0.77	5.4 ± 0.52	0.59
Hypertension (%)	34.2	38.5	0.78
Dyslipidemia (%)	26.8	35.3	0.52
Macroadenoma (%)	74.7	100	0.11
Tumor diameter (mm)	14.1 ± 5.7	24.8 ± 6.5	< 0.01**
Random GH (ng/mL)	12.1 [13.3]	0.2 [0.5]	< 0.01**
IGF-I SDS	6.6 ± 2.7	−0.2 ± 1.5	< 0.01**
PRL (ng/mL)	12.6 [13.8]	14.5 [16.1]	0.99
ACTH (pg/mL)	32.2 [20.8]	30.0 [25.1]	0.88
Cortisol (μg/dL)	12.6 ± 3.3	13.0 ± 3.5	0.68
TSH (μU/mL)	0.90 ± 0.55	1.25 ± 0.67	0.06
FT4 (ng/mL)	0.99 ± 0.22	0.96 ± 0.12	0.59
LH male (IU/mL)	2.1 [1.1]	3.1 [4.2]	0.29
LH female (IU/mL)	10.4 [11.7]	3.0 [10.7]	0.08
FSH male (IU/mL)	7.3 [7.3]	12.9 [8.8]	0.08
FSH female (IU/mL)	42.9 [55.1]	15.8 [36.2]	0.37
E2 (pg/mL)	7.1 [37.2]	0 [0]	0.12
T (ng/mL)	213.3 ± 78.2	280.8 ± 99.1	0.20

Data were compared by the χ^2^ test, Fischer’s exact test, Student’s *t*-test, or Mann—Whitney test, as appropriate.

**P < 0.01.

RTL, relative telomere length; Acro, acromegaly; NFPA, non-functioning pituitary adenoma; E2, estradiol; T, testosterone. E2 and T were measured in female and male, respectively.

To investigate the factors associated with telomere length in acromegaly, we evaluated the relationship between RTL and various clinical indices (Fig 1B–1I). In line with the previous reports, there was an inverse correlation between age and RTL (Fig 1B), although it was not statistically significant (*R* ^*2*^ = 0.056, *P* = 0.12), probably because of the relatively small sample size. There were no correlations between BMI, tumor diameter, random GH levels, nadir GH levels, or, IGF-I SDS, and RTL (Fig 1C and 1E–1H). Interestingly, there was a significant negative correlation between disease duration and RTL (Fig 1D; *R* ^*2*^ = 0.210, *P* = 0.003). After adjusting for the effect of age on RTL using multiple regression analysis, there remained a significant negative correlation between disease duration and RTL (β = −0.017, *P* = 0.04). RTL did not differ with the sex, smoking, or the presence of diabetes, hypertension, or dyslipidemia in patients with acromegaly (Fig 1I). Patients with hypertension were older than those without it (47.7 ± 12.3 v.s. 56.6 ± 8.6 years, *P* = 0.02), whereas there were no significant differences between these groups in age, sex, smoking habit, diabetes, and dyslipidemia. Since age is well known factor which influence telomere length, we re-analyzed with adjusting the effect of age using analysis of covariance (ANCOVA); however, there was no significant difference in telomere length between hypertensive and non-hypertensive patients (0.63 ± 0.28 v.s. 0.63 ± 0.26, *P* = 0.82) (S2 Fig).

### Telomere length in human fibroblasts treated with GH or IGF-I

**Fig 2:**
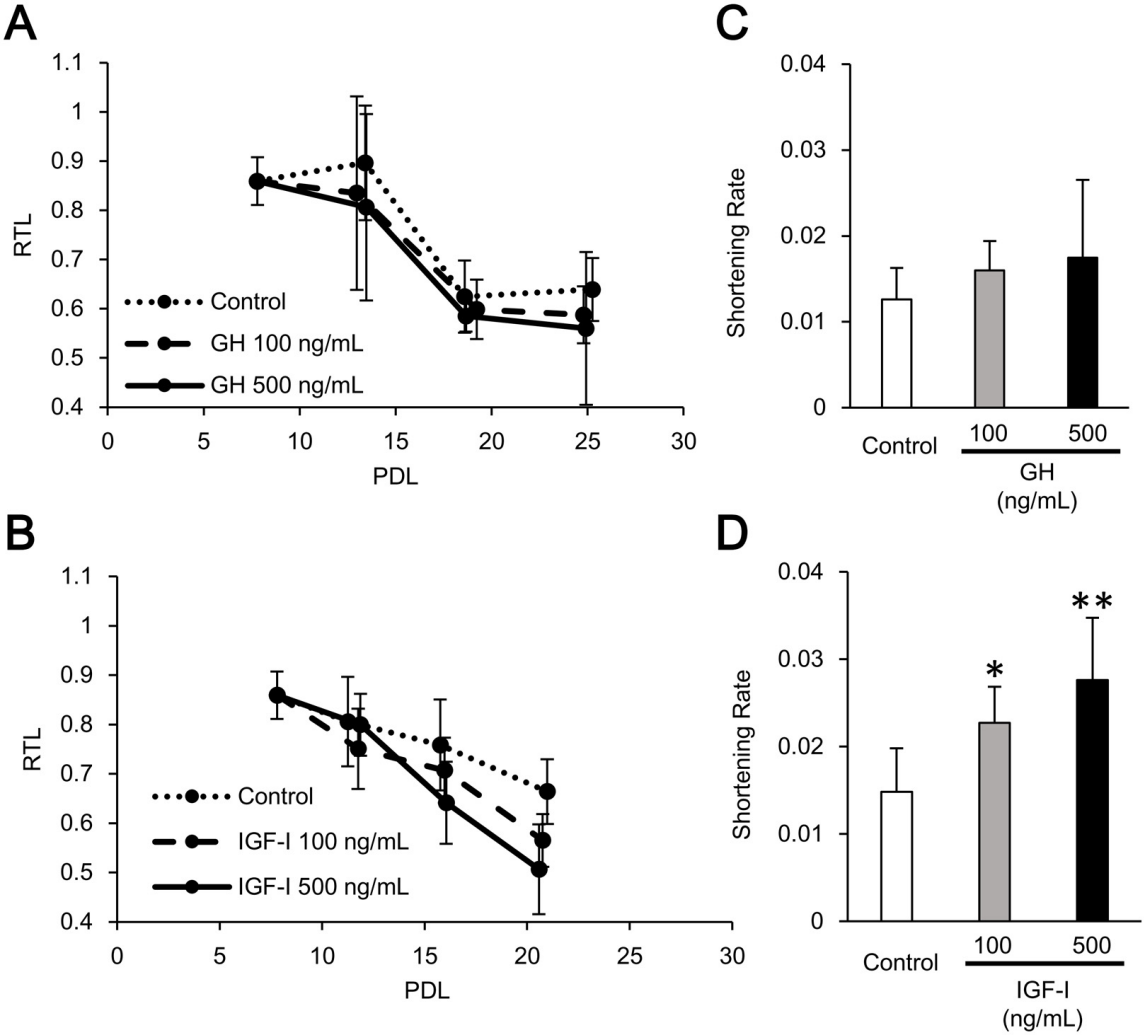
Telomere length in human fibroblasts with GH and IGF-I treatment. **A, B,** Longitudinal changes in relative telomere length in human fibroblasts treated with GH and IGF-I. **C, D,** Telomere shortening rate in human fibroblasts treated with GH and IGF-I. All data are expressed as mean ± standard deviation. Data were compared using one-way analysis of variance followed by post hoc Fisher’s least significant difference test. *P < 0.05, **P < 0.01. PDL, population doubling levels; RTL, relative telomere length.

To explore the underlying mechanisms of telomere shortening in acromegaly, we analyzed the effect of GH or IGF-I treatment on telomere length in cultured human skin fibroblasts. We examined telomere length in logarithmic growth phase. Telomere length gradually shortened in each cell (Fig 2). Intriguingly, IGF-I-treated cells showed shorter telomeres than GH- or vehicle-treated cells at a PDL of 20 (Fig 2A and 2B). The telomere shortening rate, which is defined as the changes in RTL normalized to the number of cell division ($\left|\frac{\Delta RTL}{\Delta PDL}\right|$), was significantly higher in IGF-I-treated cells than in the control cells, while there were no significant differences observed between the GH-treated and control cells (Fig 2C and 2D), indicating that IGF-I, and not GH, shortened telomeres *in vitro*.

**Fig 3:**
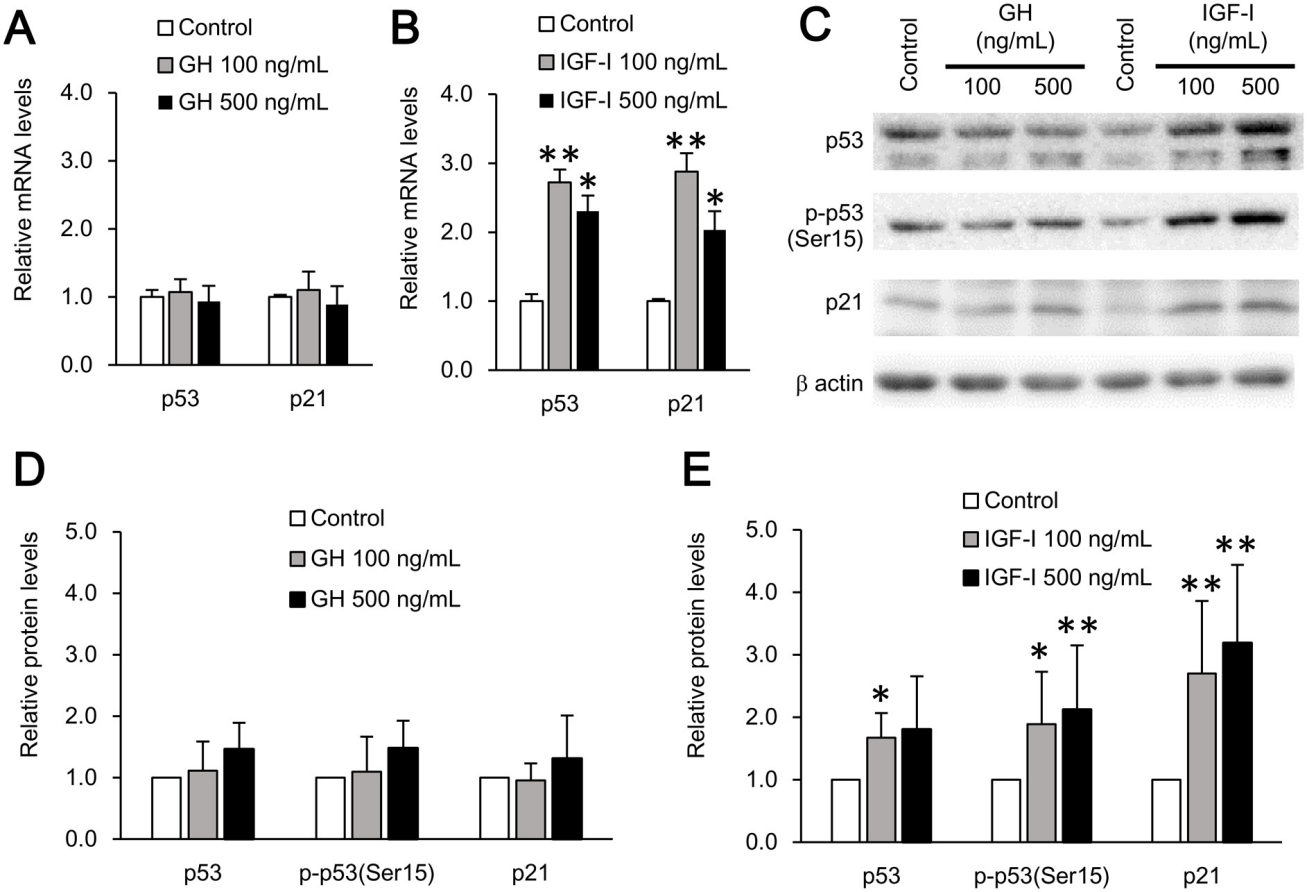
IGF-I enhanced p53-p21 pathway in human fibroblasts. **A, B,** p53 and p21 mRNA expression in human fibroblasts treated with GH or IGF-I for 27 days (PDL of 20–21). The expressions were measured using quantitative RT-PCR and normalized to β-actin. **C,** Immunoblotting analysis of p53, serine-phosphorylated-p53 (p-p53), and p21 proteins in human fibroblasts treated with GH or IGF-I for 27 days (PDL of 20–21). Anti-p-p53 protein at serine 15 antibody was used for the detection of p-p53. **D, E,** Densitometric analysis of p53, p-p53, and p21 protein levels normalized to β-actin. All data are expressed as mean ± standard deviation. Data were compared using one-way analysis of variance followed by post hoc Fisher’s least significant difference test. *P < 0.05, **P < 0.01; PDL, population doubling levels.

**Fig 4:**
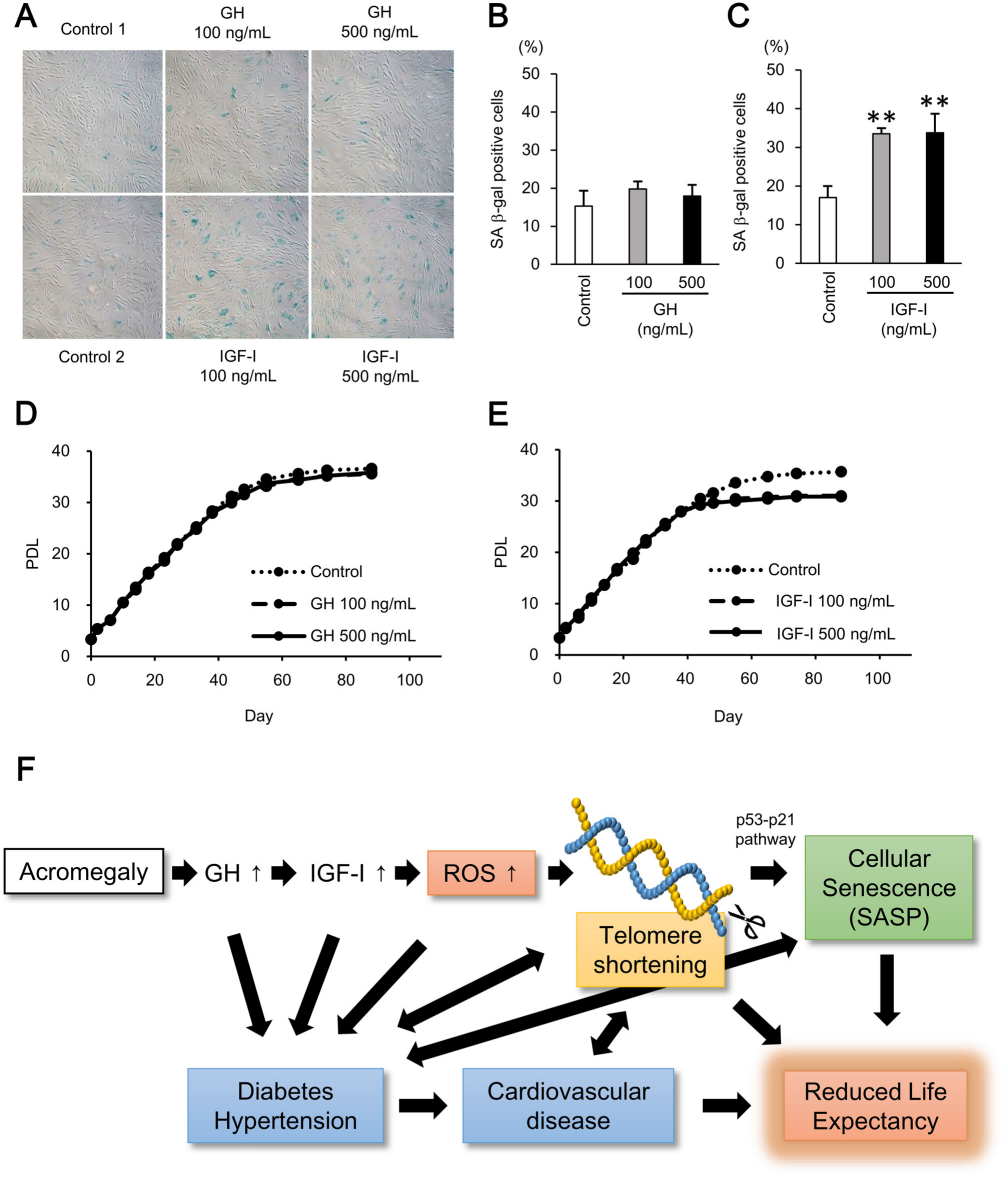
IGF-I induced cellular senescence in human fibroblasts. **A,** Senescence-associated β-galactosidase staining in human fibroblasts treated with GH or IGF-I for 38 days (PDL of 28–29). **B, C,** Rate of senescence-associated β-galactosidase activity (SA β-gal)-positive cells. **D, E,** Cell growth curves of human fibroblasts treated with GH or IGF-I. **F,** Proposed mechanistic model, in which excess secretion of GH and IGF-I causes increased comorbidities and mortality in patients with acromegaly. Data are expressed as mean ± standard deviation. Data were compared using χ^2^ test followed by Tukey’s honestly significant difference test. **P < 0.01; PDL, population doubling levels.

It has been reported that telomere shortening causes cellular senescence via the p53-p21 pathway [21] and IGF-I enhances cellular senescence in confluent human fibroblasts [20]. We explored whether IGF-I itself induces cellular senescence using human fibroblasts. Our study design is illustrated in S1A Fig. Fibroblasts treated with GH or IGF-I for 10 days (PDL of 10–11) showed comparable expression of p53 and p21 to vehicle-treated cells at the mRNA and protein levels (S1B–S1F Fig). However, after stimulation of GH or IGF-I for 27 days (PDL of 21–22), IGF-I, but not GH, treatment significantly increased the expression of p53 and p21 mRNA (Fig 3A and 3B). IGF-I also increased p53, serine-phosphorylated p53, and p21 protein levels (Fig 3C–3E). Considering the number of PDL, these results suggest that after the telomere shortening caused by IGF-I treatment, induction of the p53-p21 pathway occurred. Comparably, not GH but IGF-I increased senescence-associated β-galactosidase (SA β-gal) activity at day 38 (PDL of 28–29) (Fig 4A–4C). Regarding the cell growth curve, as previously described, IGF-I-treated cells proliferated faster than control cells in the first 10 days of stimulation, whereas GH did not show any such growth effect (S3A and S3B Fig). However, the effect on proliferation of IGF-I did not last more than 2 weeks and eventually each cell showed similar doubling time in the logarithmic growth phase (Fig 4D and 4E). Intriguingly, fibroblasts treated with IGF-I reached the Hayflick limit approximately 5 PDL earlier than GH- or vehicle-treated cells (Fig 4D and 4E), clearly demonstrating that IGF-I induces cellular senescence. Furthermore, we examined the effect of GH or IGF-I on IL-6 mRNA expression, because it has been reported that cellular senescence is associated with an inflammatory response through production of inflammatory cytokines or chemokines (senescence-associated secretary phenotype; SASP) [22]. Indeed, IGF-I-treated cells showed increased mRNA expression of IL-6 (S4 Fig).

## Discussion

In the present study, we demonstrated that patients with acromegaly exhibited shorter telomeres than the age-, sex-, smoking-, and diabetes-matched patients with NFPA in peripheral leukocytes. In addition, there was a significant negative correlation between telomere length and disease duration in acromegalic patients. These data suggest that excessive secretion of GH or IGF-I is associated with telomere shortening.

Accumulating evidence demonstrates a clear link between telomere length and mortality in various conditions; for example, a pioneering study by Cawthon et al. reported that subjects aged 60 years or older with shorter telomeres revealed a significant increase in the all-cause mortality rate compared to those with normal telomere lengths [6]. Furthermore, telomere shortening is reportedly associated with increased age-related diseases such as cardiovascular disease, cerebrovascular disease, hypertension, and diabetes [11, 23, 24]. It is well known that patients with acromegaly exhibit increased morbidity of age-related disease such as cardiovascular disease, cerebrovascular disease, hypertension, and diabetes, and premature mortality [25, 26]. Taken together with the present data, it is speculated that the shortened telomeres in acromegalic patients are closely associated with these pathological conditions, although it remains unknown whether the shortened telomeres play a causal role.

When telomeres become critically short, they become dysfunctional and activate the DDR pathway that generally is caused by double-strand breaks [27]. The resulting signaling cascade induces apoptosis and/or a permanent cell cycle arrest, and cellular senescence via the p53-p21 pathway. In the present report, we have demonstrated that not GH but IGF-I induced telomere shortening and cellular senescence in human fibroblasts. Recently, it has emerged that cellular senescence plays important roles not only in cancer protection but also in the development of age-related diseases such as diabetes and atherosclerosis, which are often associated with acromegaly, and aging itself [28, 29]. The onset of these age-related diseases is reportedly associated with SASP [22]. In line with this, IGF-I-treated cells showed an increased IL-6 mRNA expression, which is known as SASP-related cytokine. In conjunction with the effect of telomere shortening, the resulting cellular senescence may be associated with the increased morbidity and mortality in acromegalic patients.

In this study, we clearly demonstrated that not GH but IGF-I induced telomere shortening and cellular senescence in human skin fibroblasts. Generally, GH induces IGF-I production and the IGF-I exerts various actions in many cell lines, but the effect of GH-induced IGF-I is mainly depending on the ability to produce the autocrine IGF-I. Interestingly, it has been reported that human skin fibroblast has a relatively low ability to produce IGF-I by GH stimulation. For example, the concentration of IGF-I in the supernatant of the GH (20 ng/mL, 72hrs)-stimulated- fibroblasts was as low as 0.48 ± 0.09 ng/mL of IGF-I [30], which is much lower concentration of IGF-I than those we used in this study. This could explain the discrepancy in the effect on inducing telomere shortening and cellular senescence between GH and IGF-I stimulation and a presence of the IGF-I specific effect. It is also speculated that *in vivo* condition, GH induces enough amount of IGF-I mainly in the liver and the endocrine IGF-I may cause telomere shortening.

The underlying mechanisms how IGF-I induces telomere shortening and subsequent cellular senescence remains unclarified. It has been reported that various factors including ROS, defects in the telomere repair system, inflammatory reactions, and increased cellular turnover, cause telomere shortening [21]. Intriguingly, oxidative stress was enhanced both in GH-transgenic rats and patients with acromegaly [31]. In addition, it has been reported that IGF-I enhances ROS-p53 pathway and subsequent cellular senescence in cultured cells with a confluent status [20]. Bayram et al. also reported that patients with acromegaly exhibited increased oxidative stress and DNA damage [32]. Furthermore, the causal role of increased oxidative stress in telomere shortening has been reported. Human fibroblasts cultured under 40% oxygen, in which oxidative stress is increased, exhibit an accelerated rate of telomere shortening [33], and inhibition of the glutathione-dependent antioxidant system results in telomere shortening and senescence in human endothelial cells [34]. On the other hand, it is reported that IGF-I stimulates telomerase activity in cord blood mononuclear cells treated with phytohaemaglutinin (T-cell stimulating agent). However, without phytohaemaglutinin, IGF-I did not enhance telomerase activity [35]. Also, IGF-I activates telomerase in several cancer cell lines [36]; however, this might be the cell specific effect especially in cancer cells and the effect of IGF-I on telomerase has not been reported in normal somatic cells, including lymphocytes or skin fibroblasts which have little telomerase activity. Taken together, although further investigation is needed, we speculate that the increased oxidative stress may lead to the telomere shortening in acromegalic patients. Accordingly, we propose a schematic model that explains the pathological condition in acromegaly (Fig 4F). Increased IGF-I secretion in acromegaly induces ROS, telomere shortening, and cellular senescence. These factors can be causally associated with comorbidities such as diabetes, hypertension, and cardiovascular disease, and increased mortality.

It is well known that the GH-IGF-I axis plays an essential role in the regulation of aging and longevity [37]. Genetically modified mice with reduced activity in the GH-IGF-I axis show increased life span and are protected from cancer and diabetes, two major aging-related morbidities [38, 39]. A cohort study of the Laron syndrome, in which GH receptor (GHR) is deficient and serum IGF-I levels are extremely low, demonstrated that the prevalence of cancer and diabetes was drastically decreased. Intriguingly, serum from subjects with GHR deficiency reduced DNA breaks but increased apoptosis in human mammary epithelial cells treated with hydrogen peroxide. These cells showed a decreased IGF-I signaling and the effect of the serum was completely reversed by IGF-I treatment, indicating that IGF-I signaling is responsible for these effects [40]. Taken together with the present data, although several pathways including mTOR-S6K, Foxo, oxidative stress resistance, and chronic inflammation are potentially underlying mechanisms, in which GH-IGF-I regulates aging and longevity [41], it is suggested that the telomere shortening and subsequent cellular senescence by IGF-I might be involved.

This study has several limitations. One was the inherent difficulty in the selection of control subjects. Because many factors affect telomere length, it is important to exclude confounding factors as possible; we chose age-, sex-, smoking-, and diabetes-matched patients with NFPA. Although it was not significant, there was a tendency to increase the prevalence of diabetes in patients with acromegaly, which might affect telomere lengths. Also, this study was based on a relatively small number of patients and there is a difference in the number of patients between acromegaly and NFPA group, which could cause a selection bias. Nonetheless, we demonstrated the significant difference in telomere lengths between patients with acromegaly and NFPA. Furthermore, the significant association between the disease duration and telomere length strongly supports the hypothesis that a long exposure to IGF-I could lead to DNA damage and telomere shortening. Although we have demonstrated telomere shortening in peripheral leukocytes in patients with acromegaly, it is unclear whether the same condition occurs in the other tissues. However, it has been reported that telomere shortening has been observed not only in peripheral leukocytes but also in cardiomyocytes in patients with heart failure [42] and many studies using peripheral leukocytes clearly showed the significance of telomere shortening in various diseases [43], suggesting the validity of this study.

In conclusion, we have demonstrated that patients with acromegaly exhibit shortened telomeres. The *in vitro* analysis shows that IGF-I reduces telomere length and induces cellular senescence. Although further investigation is necessary, the present data can explain, in part, the underlying mechanisms by which acromegaly exhibits the increased morbidity and mortality in association with the excess secretion of IGF-I.

## Supporting Information

S1 Fig Expression of p53 and p21 in human fibroblasts treated with GH or IGF-I. **A,** Study design; Human fibroblasts were treated with 100 and 500 ng/mL of GH, IGF-I, or vehicle. At day 10 and 27, quantitative RT PCR (qRT-PCR) and immunoblotting (IB) were performed. At day 38, senescence-associated β-galactosidase staining (SA β-gal) was performed. **B, C**, p53 and p21 mRNA expression in human fibroblasts treated with GH or IGF-I for 10 days (PDL of 10–11). The expression levels were measured using qRT-PCR and normalized to β-actin. **D,** Immunoblotting analysis of p53, serine-phosphorylated-p53, and p21 proteins in human fibroblasts treated with GH or IGF-I for 10 days (PDL of 10–11). Anti-phosphorylated p53 protein at serine 15 antibody was used for the detection of p-p53. **E, F,** Densitometric analysis of p53, p-p53, and p21 protein levels normalized to β-actin. Data were compared using one-way analysis of variance followed by post-hoc Fisher’s least significant difference test or Kruskal—Wallis test followed by post-hoc Scheffe test. *P < 0.05, **P < 0.01; PDL, population doubling levels.
(TIF)

S2 Fig Comparison of relative telomere length for the clinical indices with adjusting for the age in acromegaly. Relative telomere length was compared in patients with acromegaly after adjusting the effect of age on telomere length using analysis of covariance (ANCOVA).
(TIF)

S3 Fig Cell growth curves of human skin fibroblasts treated with GH or IGF-I (early phase of the culture). **A,** GH-treated cells. **B,** IGF-I-treated cells.
(TIF)

S4 Fig IGF-I increased senescence-associated secretary phenotype cytokine expression. **A, B,** IL-6 mRNA expression in human fibroblasts treated with GH or IGF-I for 27 days (PDL of 20–21). The expression levels were measured using qRT-PCR and normalized to β-actin.
(TIF)

S1 Table Primer sequences and concentrations used for telomere measurement. (DOC)

S2 Table Primer sequences used in quantitative reverse transcription PCR. (DOC)
