# Supplementary figures and images for: Accelerated Telomere Shortening in Acromegaly; IGF-I Induces Telomere Shortening and Cellular Senescence
Source: PLoS One. 2015 Oct 8;10(10):e0140189. doi: 10.1371/journal.pone.0140189 (PMC4598111; doi:10.1371/journal.pone.0140189)

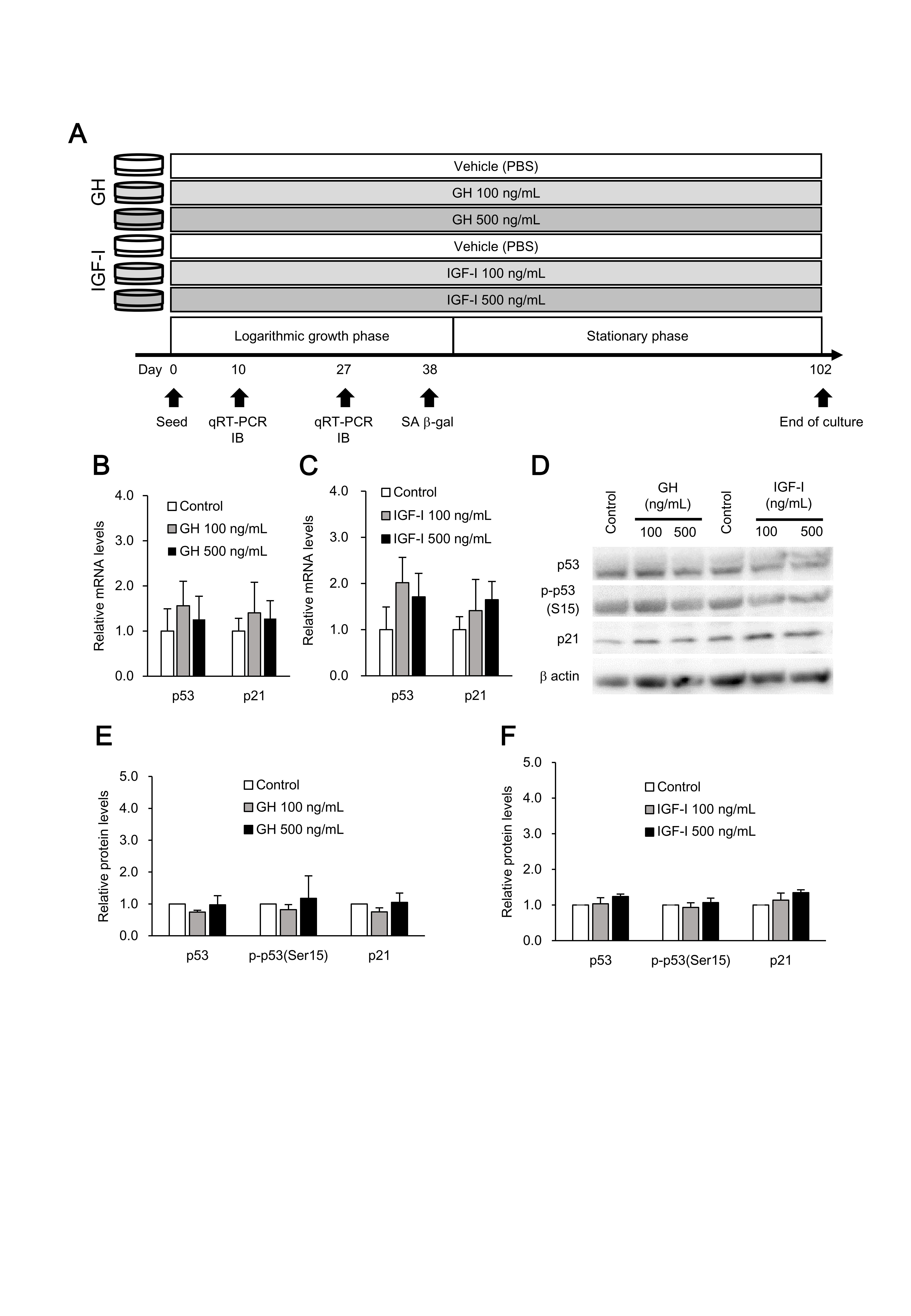

Supplement: S1 Fig — A, Study design; Human fibroblasts were treated with 100 and 500 ng/mL of GH, IGF-I, or vehicle. At day 10 and 27, quantitative RT PCR (qRT-PCR) and immunoblotting (IB) were performed. At day 38, senescence-associated β-galactosidase staining (SA β-gal) was performed. B, C, p53 and p21 mRNA expression in human fibroblasts treated with GH or IGF-I for 10 days (PDL of 10–11). The expression levels were measured using qRT-PCR and normalized to β-actin. D, Immunoblotting analysis of p53, serine-phosphorylated-p53, and p21 proteins in human fibroblasts treated with GH or IGF-I for 10 days (PDL of 10–11). Anti-phosphorylated p53 protein at serine 15 antibody was used for the detection of p-p53. E, F, Densitometric analysis of p53, p-p53, and p21 protein levels normalized to β-actin. Data were compared using one-way analysis of variance followed by post-hoc Fisher’s least significant difference test or Kruskal—Wallis test followed by post-hoc Scheffe test. *P < 0.05, **P < 0.01; PDL, population doubling levels. (TIF) [file pone.0140189.s001.tif]

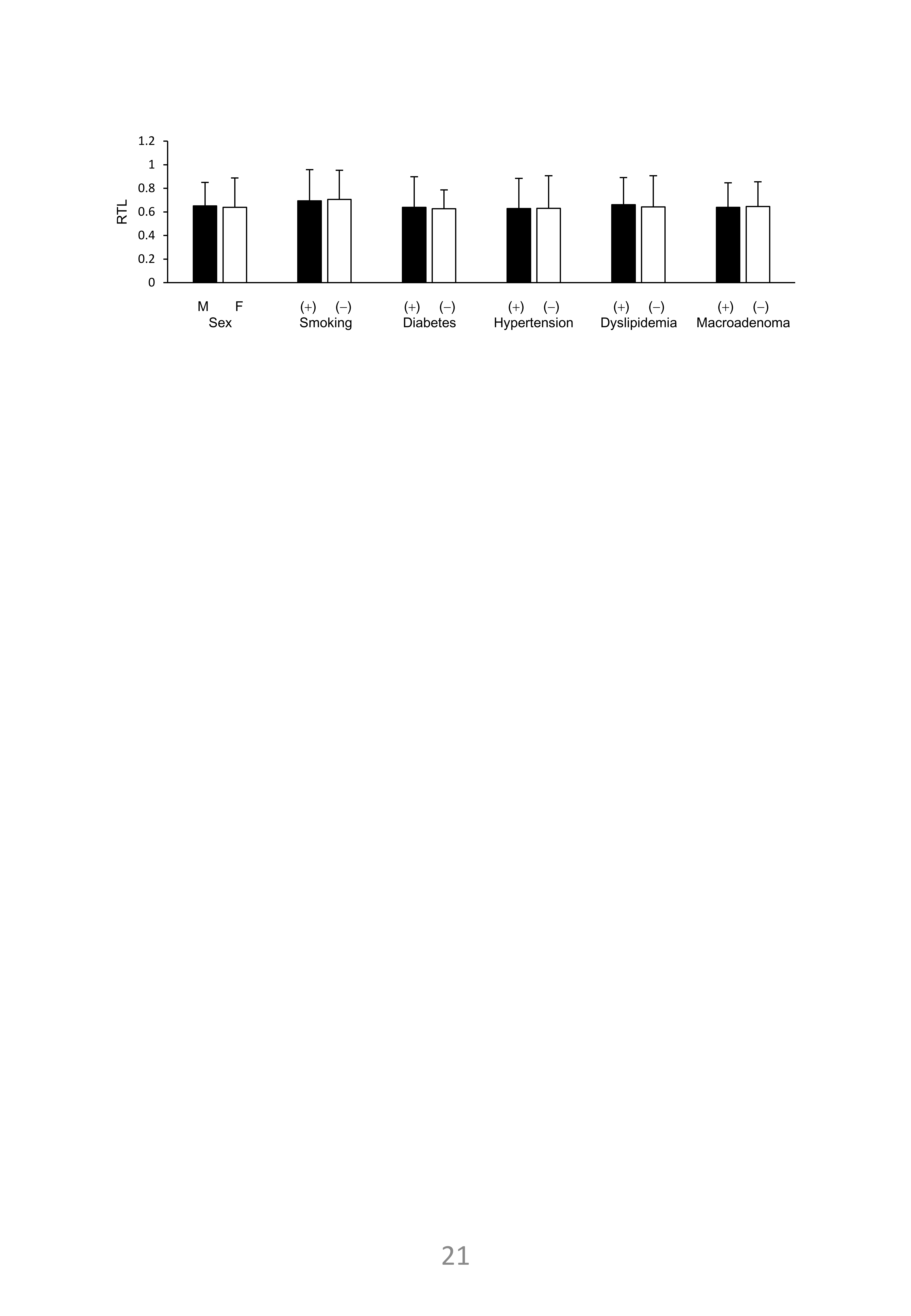

Supplement: S2 Fig — Relative telomere length was compared in patients with acromegaly after adjusting the effect of age on telomere length using analysis of covariance (ANCOVA). (TIF) [file pone.0140189.s002.tif]

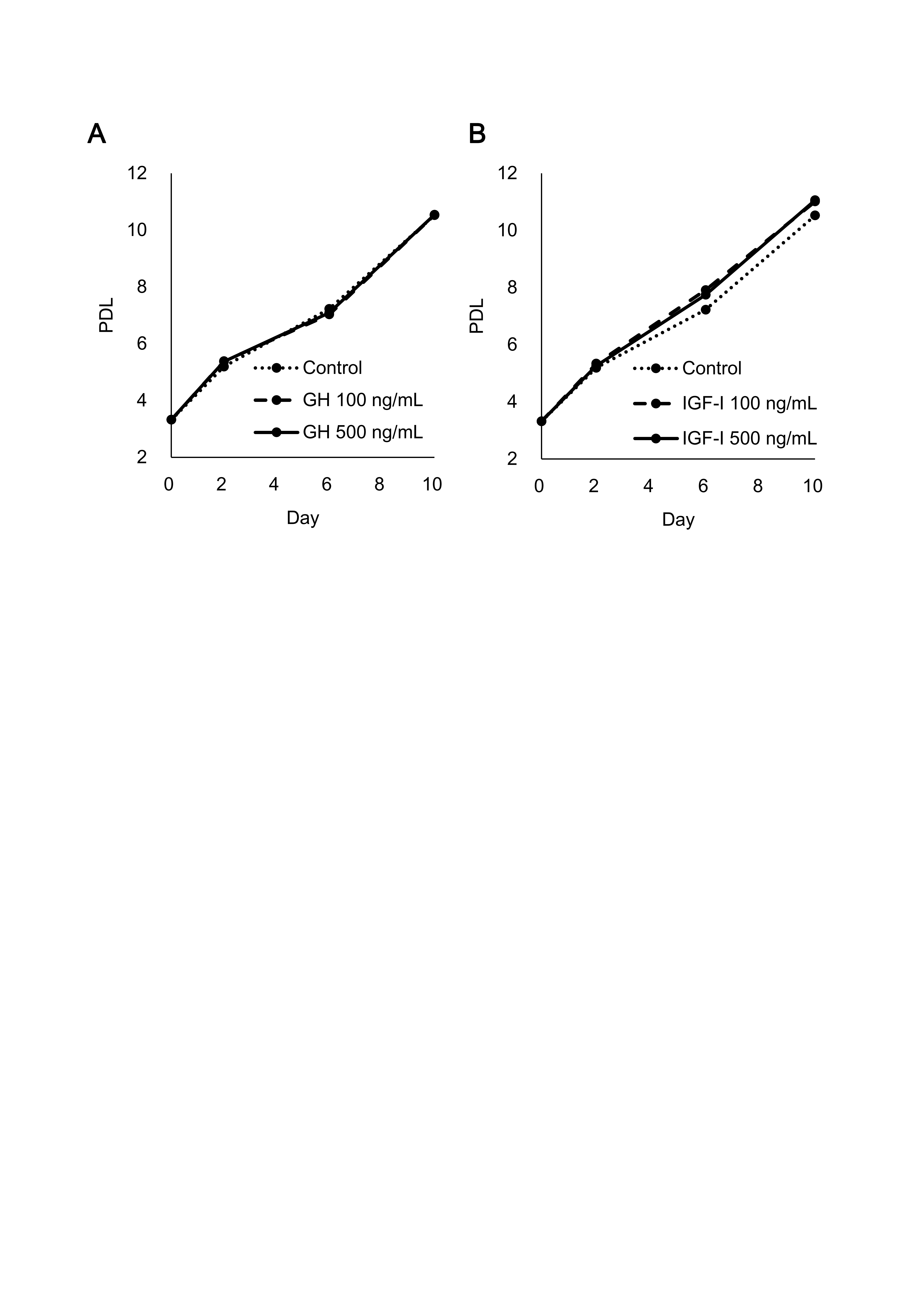

Supplement: S3 Fig — A, GH-treated cells. B, IGF-I-treated cells. (TIF) [file pone.0140189.s003.tif]

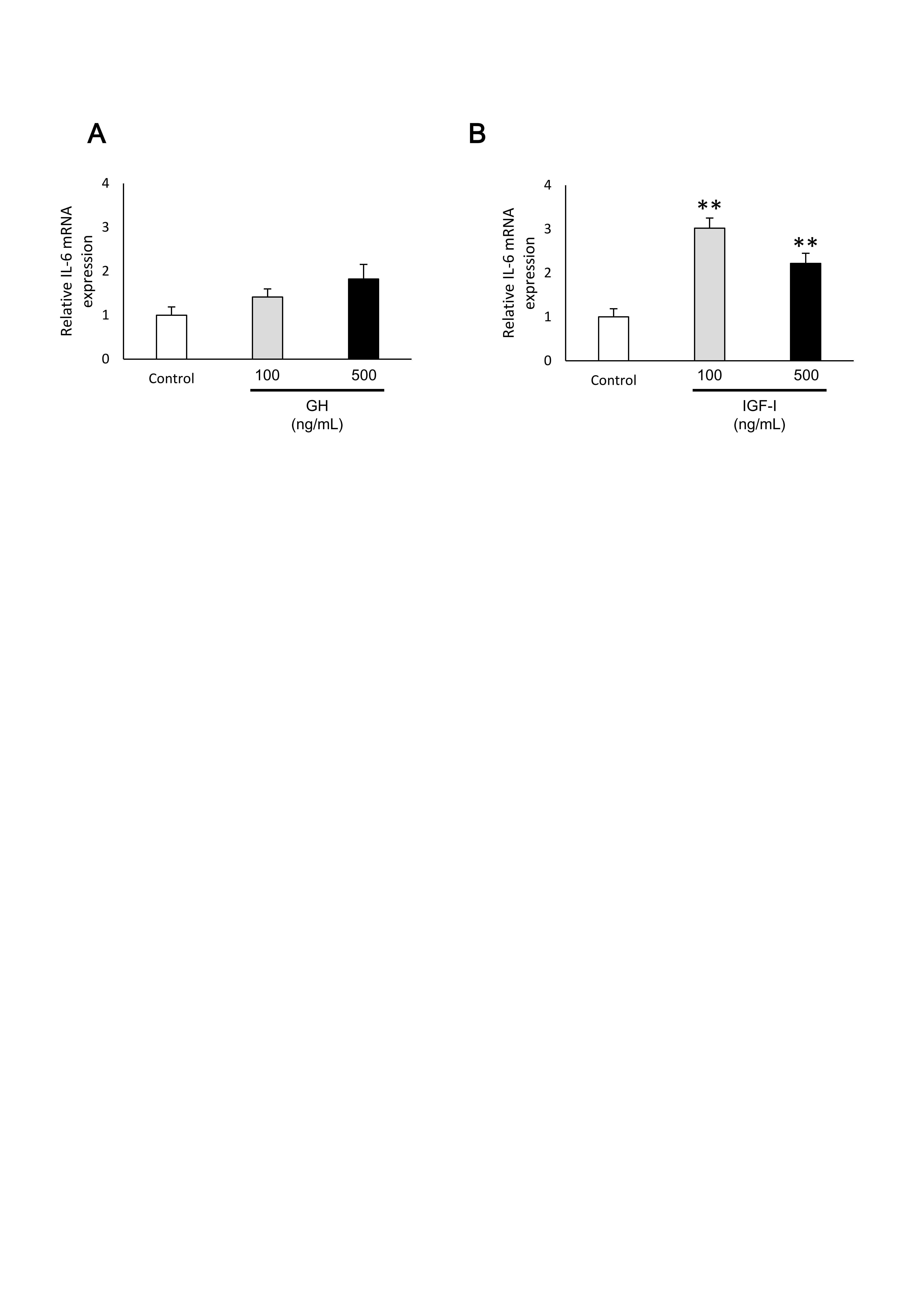

Supplement: S4 Fig — A, B, IL-6 mRNA expression in human fibroblasts treated with GH or IGF-I for 27 days (PDL of 20–21). The expression levels were measured using qRT-PCR and normalized to β-actin. (TIF) [file pone.0140189.s004.tif]
