# Supplementary material for: Accelerated Telomere Shortening in Acromegaly; IGF-I Induces Telomere Shortening and Cellular Senescence
Source: PLoS One. 2015 Oct 8;10(10):e0140189. doi: 10.1371/journal.pone.0140189 (PMC4598111; doi:10.1371/journal.pone.0140189)
Supplement: S1 Table — (DOC) [file pone.0140189.s005.doc]

|  | Group | Age, sex | Reason for exclusion |
| --- | --- | --- | --- |
| 1 | Acro | 45 yo, F | Malignancy (thyroid cancer) and thyroxin replacement therapy |
| 2 | Acro | 24 yo, M | GH suppression to less than 1 ng/mL during 75g OGTT |
| 3 | Acro | 61 yo, M | GH suppression to less than 1 ng/mL during 75g OGTT |
| 4 | Acro | 48 yo, M | GH suppression to less than 1 ng/mL during 75g OGTT |
| 5 | Acro | 23 yo, F | GH suppression to less than 1 ng/mL during 75g OGTT |
| 6 | Acro | 45 yo, M | GH suppression to less than 1 ng/mL during 75g OGTT |
| 7 | Acro | 50 yo, M | GH suppression to less than 1 ng/mL during 75g OGTT |
| 8 | Acro | 30 yo, M | GH suppression to less than 1 ng/mL during 75g OGTT |
| 9 | Acro | 50 yo, F | Previous pituitary surgery |
| 10 | Acro | 34 yo, F | Previous pituitary surgery |
| 11 | Acro | 54yo, F | Previous pituitary surgery |
| 12 | Acro | 27yo, F | Sex steroid replacement therapy |
| 13 |  |  |  |
| 14 |  |  |  |
| 15 |  |  |  |
| 16 | NFPA |  |  |
| 17 |  |  |  |
| 18 |  |  |  |
| 19 |  |  |  |
| 20 |  |  | Previous pituitary surgery |
| 21 |  |  | Previous pituitary surgery |
| 22 |  |  |  |
