# Supplementary material for: Accelerated Telomere Shortening in Acromegaly; IGF-I Induces Telomere Shortening and Cellular Senescence
Source: PLoS One. 2015 Oct 8;10(10):e0140189. doi: 10.1371/journal.pone.0140189 (PMC4598111; doi:10.1371/journal.pone.0140189)
Supplement: S2 Table — (DOC) [file pone.0140189.s006.doc]

| Primer | Sequence (5’ to 3’) |
| --- | --- |
| p53 | GTT CCG AGA GCT GAA TGA GG |
|  | TTA TGG CGG GAG GTA GAC TG |
| p21 | GGA AGA CCA TGT GGA CCT GT |
|  | GGC GTT TGG AGT GGT AGA AA |
| IL-6 | GTA GCC GCC CCA CAC AGA CAG CC |
|  | GCC ATC TTT GGA AGG TTC AGG |
| β actin | GCA AGC AGG AGT ATG ACG AG |
|  | CAA ATA AAG CCA TGC CAA TC |
